# Transgender adults, gender-affirming hormone therapy and blood pressure: a systematic review

**DOI:** 10.1097/HJH.0000000000002632

**Published:** 2020-11-05

**Authors:** Paul J. Connelly, Anna Clark, Rhian M. Touyz, Christian Delles

**Affiliations:** Institute of Cardiovascular and Medical Sciences, University of Glasgow, Glasgow, UK

**Keywords:** blood pressure, estrogen, testosterone, transgender

## Abstract

Gender-affirming hormone therapy (GHT) is utilized by people who are transgender to align their secondary sex characteristics with their gender identity. Data relating to cardiovascular outcomes in this population are limited. We aimed to review the impact of GHT on the blood pressure (BP) of transgender individuals.

**Methods:**

We searched PubMed/MEDLINE, SCOPUS and Cochrane Library databases for articles published relating to the BP of transgender adults commencing GHT. Methodological quality was assessed via the ‘Quality Assessment Tool for Before–After (Pre–Post) Studies with No Control Group’.

**Results:**

Six hundred articles were screened, of which 14 studies were included in this systematic review encompassing 1309 individuals (∼50% transgender men and women) treated with GHT between 1989 and 2019. These articles were all pre–post observational studies without control groups. Mean ages ranged between 23.0–36.7 years (transgender men) and 25.2–34.8 years (transgender women). Interventions were diverse and included oral, transdermal and injectable hormonal preparations with 4 months to 5 years follow-up. Most studies in transgender men did not demonstrate a change in BP, whereas transgender women on GHT demonstrated both increases and decreases in SBP. These studies were heterogenous with significant methodological limitations and only two were determined to have a good quality rating.

**Conclusion:**

There is currently insufficient data to advise the impact of GHT on BP in transgender individuals. Better quality research is essential to elucidate whether exogenous sex hormones modulate BP in transgender people and whether this putative alteration infers poorer cardiovascular outcomes.

## INTRODUCTION

Transgender people experience gender dysphoria due to incongruence between their gender identity and the sex they were assigned at birth [1]. Gender-affirming hormone therapy (GHT), including testosterone, estrogen, gonadotropin-releasing hormone (GnRH) analogues and antiandrogens, aims to align the characteristics of transgender people with their gender identity [1].

The recent substantive increases in population prevalence of transgender people requires the implementation of evidence-based guidance to better protect the health of this population [2–4]. However, due to a paucity of epidemiological and mechanistic data, there is considerable uncertainty surrounding the impact of GHT on the cardiovascular health of transgender individuals [5–7]. Existing data suggest that the use of estrogens in transgender women confers an increased risk of myocardial infarction and ischemic stroke. Conversely, transgender men receiving testosterone lack any consistent evidence of an increased risk of cardiovascular or cerebrovascular disease [7]. However, in the absence of randomized controlled trials or comprehensive prospective longitudinal studies, ambiguity remains regarding whether such risk exists, and by which interventions this risk can be ameliorated.

Consequently, international guidelines, set out by the Endocrine Society and World Professional Association for Transgender Health, have been cautious in their recommendations regarding modifiable risk factors for cardiovascular disease in this population [8,9]. Hypertension remains the most important modifiable risk factor for the development of cardiovascular disease. Therefore, these organizations have not unreasonably recommended that blood pressure (BP) is monitored in transgender people receiving GHT. However, these guidelines do not provide any indication of the effect size of this treatment on BP, treatment targets for this patient group, or the levels of evidence for this recommendation.

A comprehensive overview is therefore required to assess the impact of GHT on the BP. Our aim was to perform a systematic review of the relationship of exogenous sex hormones and SBP and DBP in transgender men and women commencing treatment.

## METHODS

### Eligibility criteria

We included randomized trials and observational studies of transgender individuals who used GHT regardless of gender-reassignment surgery status. Eligible studies included transgender men prescribed testosterone and transgender women prescribed estrogen, antiandrogens (cyproterone acetate, finasteride or spironolactone) or GnRH agonists. Studies were included regardless of dose of GHT. Studies were required to provide a comparison of BP before and after/during treatment for a minimum of 3 months. Articles with participants under the age of 16 were excluded in addition to review articles, commentaries and letters that did not contain original data. BP measurements could include manual, automated or ambulatory recordings. Many transgender individuals receiving GHT may be gender non-binary (e.g. their gender identity or expression does not confirm to typical binary gender), and may not consider themselves as simply men or women [3]. However, for the purposes of this systematic review, transgender people treated with estrogen (with or without antiandrogens and GnRH analogues) were considered transgender women, whereas transgender individuals treated with testosterone were considered transgender men.

### Search strategy

The current systematic review adhered to the Preferred Reporting Items for Systematic Reviews guidelines [10]. PubMed/MEDLINE, SCOPUS and Cochrane Library databases were searched for articles published until 13 January 2020. The search was limited to English language articles.

The following medical subject headings were used in the search: ‘blood pressure’ AND ‘transgender’ OR ‘transgender person’ OR ‘person, transgender’ OR ‘persons, transgender’ OR ‘transgender persons’ OR ‘transmasculine’ OR ‘transfeminine’ OR ‘trans masculinization’ OR ‘trans feminization’ OR ‘gender incongruent’ OR ‘gender dysphoric’ OR ‘gender affirming’ OR ‘transsexual persons’ OR ‘person, transsexual’ OR ‘persons, transsexual’ OR ‘transsexual person’ OR ‘transgender male’ OR ‘transgender female’ OR ‘transgender man’ OR ‘transgender woman’ OR ‘trans man’ OR ‘trans woman’ OR ‘cross-sex’.

### Data extraction, outcome and quality

The literature search, data extraction and quality assessment were conducted independently by two investigators (P.J.C. and A.C.). In the case of disagreement between the two reviewers, consensus was achieved after consulting with a third reviewer (C.D.). Duplicate records were removed and study titles and abstracts were screened to identify potentially eligible articles for inclusion in the full text review. The selected articles were read in full for confirmation of eligibility and data extraction. Data extracted from these articles included name of the first author, publication year, country, number of participants, duration of the follow-up, intervention (dose and therapy), and SBP and DBP before and after the introduction of GHT.

To determine the quality of the included studies the National Institute of Health Quality Assessment Tool for Before–After (Pre–Post) Studies with No Control Group was utilized [11]. This tool is comprised of 12 questions used to assess the risk of types of bias, such as selection, reporting or observer bias. The methodological quality of included studies was assessed by two authors (P.J.C. and A.C.) separately. Each study was defined as poor, fair or good based on the answers compiled from this tool. To determine the level of agreement between assessing authors, Cohen's kappa was calculated [12]. A score less than 0.4 is considered poor, between 0.4 and 0.8 fair to substantial, and above 0.8 as almost perfect interrater agreement. A meta-analysis of uncontrolled pre–post standardized mean differences in BP was not performed as they lack independence and fail to discern between intra-individual and interventional effects [13].

## RESULTS

### Study selection and characteristics

**FIGURE 1:**
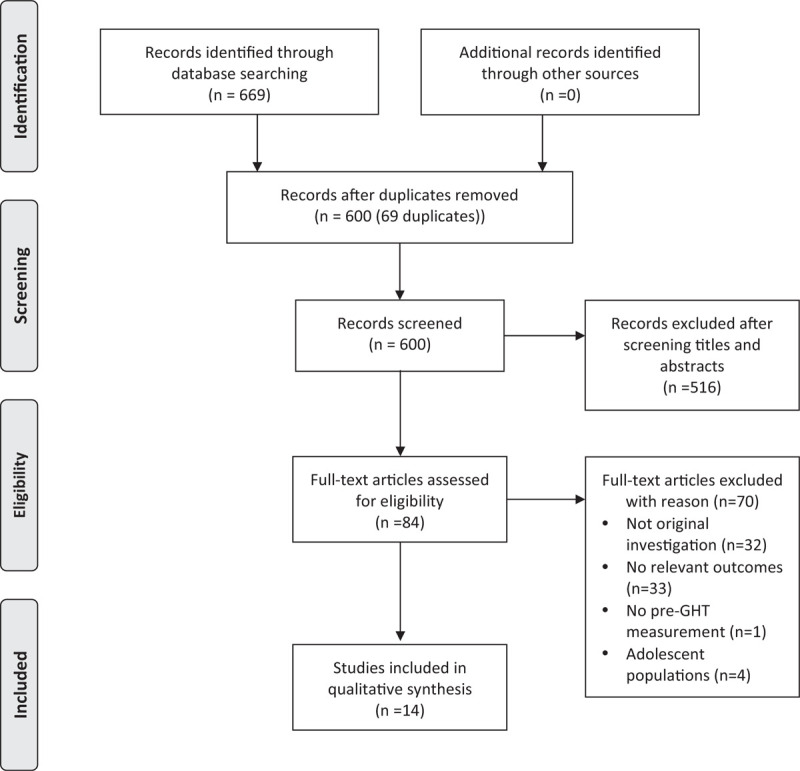
Flow diagram for study selection.

Our multidatabase literature search identified 600 nonduplicated potentially relevant records (Fig. 1). No records were identified through other resources. Following the screening of these records’ titles and abstracts, 84 full text articles were assessed for eligibility; however, 70 articles were excluded due to not being original investigations, not assessing relevant outcomes, not obtaining pre-GHT outcome measurements [14] or including adolescent populations [15–18].

Consequently, 14 studies were included in this systematic review encompassing 1309 individuals (∼50% transgender men and women) treated with GHT between 1989 and 2019. Follow-up ranged between 4 months and 5 years. 11 of the included studies were undertaken in Europe and three in North America. Mean ages ranged between 23.0–36.7 years in transgender men and 25.2–34.8 years in transgender women.

Of the included studies, all studies were pre–post observational studies without control groups. Only one study provided the device used to measure BP (BP-8800; Colin Corporation, Hayashi, Japan) [19], the position of measurement was acknowledged in four studies [19–21], and the use of multiple BP measurements mentioned in three studies [19,20,22].

### Transgender men

Between 2003 and 2019 a total of 13 studies measured the SBP and DBP of transgender men receiving GHT (Table 1). Mean follow-up time was 18.9 (SD 14.2) months (range 4–60 months). The average sample size was ∼50; however, three studies recruited less than 20 individuals [19,23,24].

**TABLE 1:** Blood pressure in testosterone exposed transgender men

						Mean difference (mmHg) [95% CI]	
Reference	Country	Follow-up (months)	Sample size, *n*	Mean age (years) (SD)	Intervention	SBP	DBP
Elbers *et al.*[19]	The Netherlands	12	17	23 (5)	250 mg IM testosterone esters/2 weeks	+1.0 [−5.1, 7.1]	−0.2 [−4.6, 4.2]
Giltay *et al.*[22]	The Netherlands	4	81	36.7 (10)	250 mg IM testosterone esters/2 weeks (*n* = 61) or oral testosterone undecanoate 160–240 mg/day (*n* = 20)	−4.6 [−8.3, −0.9]	−2.1 [−4.4, 0.2]
Mueller *et al.*[25]	Germany	12	35	29.6 (8.9)	1 g IM testosterone undecanoate/12 weeks	+4.3 [−1.5, 10.1]	+2.9 [−0.3, 6.1]
Mueller *et al.*[27]	Germany	24	45	30.4 (9.1)	1 g IM testosterone undecanoate/12 weeks	+5.2 [0.5, 9.9]	+2.8 [−0.0, 5.6]
Wierckx *et al.*[30]	Belgium, The Netherlands	12	53	24.5 (7.0)^a^	1 g IM testosterone undecanoate at baseline, then 6 weeks, then/12 weeks thereafter. If intolerant of testosterone undecanoate, 250 mg IM testosterone esters/2 weeks	+4.1 [−0.5, 8.7]^a^	+2.3 [−1.5, 6.1]^a^
Colizzi *et al.*[20]	Spain	24	43	28.8 (5.6)	250 mg IM testosterone ester/21.16 ± 3.17 days	+13.3 [9.6, 13.3]	+3.7 [0.24, 7.2]
Quirós *et al.*[31]	Spain	24	97	28.6 (8.6)	50 mg transdermal testosterone gel/day or 1 g IM testosterone undecanoate 1 g/12 weeks	+2.2 [−0.9, 5.3]	+1.5 [−1.1, 4.1]
Deutsch *et al.*[32]	USA	6	34	27 (6.9)	50–70 mg subcutaneous testosterone cypionate/week (*n* = 31), 5 g testosterone gel/day (*n* = 2) or 4 mg testosterone transdermal patch/day (*n* = 1)	+3.0 [−3.7, 9.7]^b^	−2 [−6.0, 2.0]^b^
Fernandez and Tannock [23]	USA	18	19	27 (7)	∼150 mg IM testosterone/2 weeks (type unspecified)	−2.0 [−12.6, 8.6]	−1 [−7.9, 5.9]
Vita *et al.*[24]	Italy	25.5^c^	11	25.1 (3.7)	IM testosterone enanthate (*n* = 10) or undecanoate (*n* = 1) (dose/frequency unspecified)	+6.2 [0.5, 11.8]	+3.1 [−1.9, 8.1]
Auer *et al.*[28]	Germany	12	45	27.5 (1.3)	1 g IM testosterone undecanoate/12 weeks	+7.2 [−3.1, 17.5]	+3.4 [−2.9, 9.8]
Gava *et al.*[26]	Italy	60	50	30.1 (6.1)^a^	1 g IM testosterone undecanoate at baseline, then 6 weeks, then/12 weeks thereafter (*n* = 25) or 250 mg IM testosterone enanthate/3–4 weeks (*n* = 25)	−3.0 [−6.6, 0.6]^a^	−1.5 [−4.6, 1.6]^a^
van Velzen *et al.*[21]	Belgium, The Netherlands	12	118	26.4 (9.1)	1 g IM testosterone undecanoate/12 weeks (*n* = 79) or 250 mg IM testosterone esters/2 weeks (*n* = 62) or 50 mg testosterone gel/day (*n* = 47)	+1.5 [−1.5, 4.5]^d^	+1.8 [−0.8, 4.4]^d^

95% CI, 95% confidence interval; IM, intramuscular.

a Pooled means ± SD.

b Data extracted from median and IQR.

c Mean follow-up of cohort.

d Data derived from percentage change in baseline values after adjustment for age, SD derived from 95% CI.

Various formulations of testosterone were administered in these studies including intramuscular testosterone undecanoate (*n* = 8), intramuscular testosterone esters (*n* = 7), transdermal testosterone gel (*n* = 2) or patches (*n* = 1), oral testosterone undecanoate (*n* = 1), or subcutaneous testosterone cypionate (*n* = 1). One study did not specify the formulation of intramuscular testosterone administered [23].

As demonstrated in Table 1, the effect of testosterone GHT on BP varies considerably between these studies. The majority of these studies did not demonstrate a significant change in SBP (*n* = 9), whereas three studies reported a significant increase in SBP ranging between 4.3 and 13.3 mmHg [20,25,27] and only one study reported a significant decrease in SBP (4.6 mmHg) [22]. Most studies (*n* = 10) did not report a significant change in DBP.

### Transgender women

Table 2 summarizes included studies (*n* = 10) of transgender women receiving estrogen therapy comprising of 661 individuals. These studies were undertaken between 1989 and 2019 with a mean follow-up of 16.4 (SD 7.9) months (range 6–31.7 months). The mean sample size of these studies was 66 individuals, with the largest being conducted by van Velzen *et al.*[21] of 242 individuals.

**TABLE 2:** Blood pressure in estrogen exposed transgender women

						Mean difference (mmHg) [95% CI]	
Reference	Country	Follow-up (months)	Sample size, *n*	Mean age (years) (SD)	Intervention	SBP	DBP
Prior *et al.*[29]	Canada	12	23	30.7 (6.2)	0.625 mg increasing to 2.5 mg conjugated estrogen twice daily for 3/4 weeks, followed by 10 mg medroxyprogesterone/day during weeks 3 and 4 or continuously if gonadotrophins increased, to aid in testosterone reduction or breast development, in addition 100–200 mg spironolactone/day	−7.3 [−15.3, 0.7]^a^	−2.6 [−9.3, 4.1]^a^
Elbers *et al.*[19]	The Netherlands	12	20	26 (6)	100 μg oral ethinyl estradiol and 100 mg cyproterone acetate/day	+7.2 [0.2, 14.6]	+5.7 [0.0, 11.4]
Wierckx *et al.*[30]	Belgium, The Netherlands	12	53	30.3 (14.1)^e^	<45 Years (*n* = 40) 4 mg oral estradiol valerate and 50 mg cyproterone acetate/day. >45 Years (*n* = 13) 100 μg/24 h transdermal 17β estradiol patch. If intolerant of this, 2 mg transdermal 17β estradiol gel twice daily or 4 mg estradiol valerate per day	−5.4 [−10.8, 0.0]^e^	−0.1 [−4.0, 3.8]^e^
Colizzi *et al.*[20]	Spain	24	79	30.2 (9.6)	2.12 ± 0.57 mg transdermal estradiol gel/day and 100 mg cyproterone acetate/day	+17.8 [14.4, 21.2]	+3.2 [0.3, 6.1]
Quirós *et al.*[31]	Spain	24	150	32.4 (10.1)	Oral estrogen (conjugated equine estrogens or estradiol valerate) and either cyproterone acetate or flutamide. >40 Years were recommended transdermal estrogens (dose/frequency/formulation unspecified)	+6.4 [3.5, 9.3]	+3.7 [1.4, 5.9]
Deutsch *et al.*[32]	USA	6	16	29 (9.4)	2 mg sublingual micronized 17β estradiol twice daily (*n* = 14) or 20 mg IM estradiol valerate/2 weeks (*n* = 1) or 100 μg estradiol via transdermal patch (*n* = 1) in addition to 50–100 mg spironolactone/day (*n* = 15)	−10.0 [−17.0, −2.9]^b^	−11 [−19.7, −2.3]^b^
Fernandez and Tannock [23]	USA	18	33	31 (10)	1.71 mg oral estrogen (type unspecified)/day (∼50%), transdermal estrogen (dose/type/frequency unspecified) (14%) or IM estrogen (36%) (dose/type/frequency unspecified), and 100 mg spironolactone/day	−6.0 [−13.9, 1.9]	−5.0 [−10.6, 0.6]
Vita *et al.*[24]	Italy	31.7^c^	21	25.2 (7)	2–6 mg oral estradiol valerate/day and 50–100 mg cyproterone acetate/day (if not undergoing gender reassignment surgery). Patients (*n* = 4) receiving ethylestradiol were switched to estradiol valerate. Progesterone was also utilized (*n* = 3) (type/route/frequency unspecified)	−6.1 [−11.0, −1.2]	−4.0 [−9.1, 1.1]
Auer *et al.*[28]	Germany	12	24	34.8 (1.4)	2 mg oral estradiol valerate twice daily or 100 μg transdermal 17β estradiol patch/day (if >45 years) and 50 mg cyproterone acetate/day	−6.7 [−13.9, 0.6]	−1.7 [−6.1, 2.7]
van Velzen *et al.*[21]	Belgium, The Netherlands	12	242	32.3 (12.6)	2 mg oral estradiol valerate twice daily (*n* = 144) or 100 μg transdermal estradiol patch/day (if age >45 years, *n* = 98), and 50 mg cyproterone acetate/day	−3.4 [−5.9, −0.9]^d^	−1.8 [−4.1, 0.5]^d^

95% CI, 95% confidence interval; GHT, gender-affirming hormone therapy; IM, intramuscular.

a Data included on participants not previously exposed to GHT.

b Data extracted from median and IQR.

c Mean follow-up of cohort.

d Data derived from percentage change in baseline values after adjustment for age, SD derived from 95% CI.

e Pooled means ± SD.

The doses and formulations of GHT vary significantly between studies. Oral estradiol valerate (*n* = 6), estradiol patches (*n* = 6), estradiol gel (*n* = 2), oral ethynyl estradiol (*n* = 2), oral conjugated estrogen (*n* = 2), sublingual micronized 17β estradiol (*n* = 1), intramuscular estradiol valerate (*n* = 2) and unspecified oral estrogen (*n* = 1), were utilized. Four studies provided transdermal estrogen patches rather than oral estrogen to those over the age of 40–45 years [21,28,30,31]. The most common antiandrogen was cyproterone acetate (*n* = 7), whereas spironolactone (*n* = 3) and flutamide (*n* = 1) was also utilized. No study incorporated the use of GnRH analogues while two studies utilized progestogens.

The outcomes relating to these studies are also diverse with three studies demonstrating a rise in SBP between 7.2 and 17.8 mm Hg [19,20,31] and three studies demonstrating a fall in SBP between 3.4 and 10 mm Hg [21,24,32]. DBP was increased in two studies (3.2 to 5.7 mmHg) [19,20], with no significant change reported in seven studies.

The outcomes relating to these studies are also diverse with three studies demonstrating a rise in SBP between 7.2 and 17.8 mmHg [19,20,31] and three studies demonstrating a fall in SBP between 3.4 and 10 mmHg [21,24,32]. DBP was increased in two studies (3.2 to 5.7 mmHg) [19,20], with no significant change reported in seven studies.

### Quality of evidence

Quality of methodology was assessed using the Quality Assessment Tool for Before–After Studies with no control group (Table 3). Inter-rater (P.J.C. and A.C.) agreement was 92.8% with an expected agreement of 69.4% and Cohen's kappa of 0.77 (standard error, 0.22), indicating substantial agreement. Only two studies were determined to have a good quality rating [20,21], however, as uncontrolled pre–post studies demonstrate weak evaluative designs, no study successfully fulfilled the entire quality criteria. Largely, studies did not provide power calculations or were underpowered to demonstrate significant changes in BP. Furthermore, studies did not report blinding, lacked multiple measurements before and after the introduction of GHT and comprised of significantly heterogenous groups receiving various doses, formulations and combinations of GHT treatments.

**TABLE 3:** Quality assessment of studies assessing the effect of gender-affirming hormone therapy on blood pressure in transgender individuals

Reference	Q1	Q2	Q3	Q4	Q5	Q6	Q7	Q8	Q9	Q10	Q11	Q12	Quality rating
Prior *et al.*[29]	Y	Y	Y	Y	NR	N	N	NR	Y	Y	N	NA	Fair
Elbers *et al.*[19]	Y	Y	Y	Y	NR	Y	Y	NR	Y	Y	N	NA	Fair
Giltay *et al.*[22]	Y	Y	Y	Y	N	N	Y	N	Y	Y	N	NA	Fair
Mueller *et al.*[25]	Y	Y	Y	Y	NR	Y	N	N	Y	Y	N	NA	Fair
Mueller *et al.*[27]	Y	Y	Y	Y	NR	Y	N	N	Y	Y	N	NA	Fair
Wierckx *et al.*[30]	Y	Y	Y	Y	NR	N	N	N	Y	Y	N	NA	Fair
Colizzi *et al.*[20]	Y	Y	Y	Y	Y	Y	Y	N	Y	Y	N	NA	Good
Quirós *et al.*[31]	Y	Y	Y	Y	Y	N	N	N	N	Y	N	NA	Fair
Deutsch *et al.*[32]	Y	Y	Y	Y	NR	N	Y	N	Y	Y	N	NA	Fair
Fernandez and Tannock [23]	Y	Y	Y	Y	NR	N	N	N	N	Y	N	NA	Poor
Vita *et al.*[24]	Y	Y	Y	Y	NR	N	Y	N	Y	Y	N	NA	Fair
Auer *et al.*[28]	Y	Y	Y	Y	NR	N	N	N	Y	Y	N	NA	Fair
Gava *et al.*[26]	Y	Y	Y	Y	NR	N	N	N	Y	Y	N	NA	Fair
van Velzen *et al.*[21]	Y	Y	Y	Y	Y	N	Y	N	N	Y	N	NA	Good

N, no; NA, not applicable; NR, not reported; Y, yes.

## DISCUSSION

The aim of this systematic review was to gain insights on the effects of GHT on BP of transgender men and women. This systematic review has examined the available evidence relating to BP alteration in this population. Overall, we have identified 14 studies relating to transgender people receiving GHT, who demonstrated pre and post GHT BP measurements.

Our findings demonstrate that current evidence is derived from uncontrolled pre–post studies, which are heterogenous in their recorded interventions and lack consistency in their outcomes. The majority of studies included in this systematic review demonstrate poor to fair quality, even after taking into account the limitations of uncontrolled study designs. Other common weaknesses include short follow-up durations, lack of standardization in delivery of GHT and small sample sizes, thereby limiting their power. Only one study provided information regarding the device used to measure BP (Colin, BP-8800) [19]. However, this device has been demonstrated to exhibit inconsistent aberration patterns, thereby invalidating its use in clinical research [33]. Lastly a meta-analysis is not possible as such results lack independence and cannot discern between intraindividual and GHT effects [13].

A limitation of this systematic review is that it did not include cross-sectional assessments of the development of hypertension. Asscheman *et al.*[34] observed an increase in the crude incidence of hypertension in transgender women but not men, and no difference in hypertension standardized incidence ratio (defined as >160/95 mmHg) in either groups when compared with cisgender populations. Furthermore, self-reported hypertension in the Behavioral Risk Factor Surveillance System survey has been found to be no higher than cisgender populations [35]. Results from these studies are also derived from relatively small populations and rely on outdated or self-reported hypertension definitions, thereby limiting their utility.

As many as 390 adults per 100 000 of the US population identify as transgender and this marginalized group suffer from significant health disparities [3,36]. To enact better care and evidence-based guidance for this expanding population, the collection and reporting of good quality data are imperative [1]. Randomized controlled trials reduce bias and permit the examination of the causal inference of an intervention, however, may not be permissible or ethical in this population [1]. However, the risk of bias inherent in the intrinsically weak evaluative quasiexperimental uncontrolled before–after design could be reduced by the introduction of cisgender controls (ideally both male and females per transgender individual) or an interrupted time series design, which would allow the intervention effect to be estimated accounting for the underlying trend [37]. Given the limited age groups that were assessed in these studies, research focusing on the interaction between advancing age and life-long GHT is also vital [38].

Furthermore, in line with recent guidance on BP assessment in clinic-based research, it is recommended that multiple BP readings be taken from a validated and calibrated BP device and averaged at each assessment [39]. Multiple visits with BP assessments are undertaken at baseline and during intervention follow-up, which would permit a simple interrupted time series design. The addition of ambulatory BP monitoring should be encouraged.

The majority of studies of transgender men receiving GHT did not demonstrate a significant change in BP, whereas both increases and decreases in SBP were observed in transgender women receiving hormone therapy. Mechanistically, estrogen promotes endothelium-dependent vasorelaxation via increasing nitric oxide bioavailability, expression and activation of endothelial nitric oxide synthase, increases in endothelium-derived hyperpolarizing factor and prostacyclin, and reductions in endothelin-1 [40–44]. Endothelium-independent vasodilation is achieved by modulation calcium flux in the vascular smooth muscle cells (VSMCs) [45]. Conversely, testosterone may facilitate either vasodilatation or vasoconstriction via endothelium-independent inhibition of voltage-operated calcium channels and activation of potassium channels in VSMCs, and enhancing thromboxane A2-mediated and endothelin-1-mediated vasoconstriction, respectively [46,47]. Importantly, sex hormones may also indirectly modulate vasodynamic pathways such as the renin–angiotensin–aldosterone system [48–50]. The putative relationship between estrogen and elevated BP in transgender women may highlight fundamental gaps in our understanding of the vasoconstrictive properties of this sex hormone.

However, due to the limited quality of the of the studies included in this review, there is insufficient data to determine meaningful conclusions regarding the effect of estrogen or testosterone-based GHT on the SBP or DBPs of transgender individuals or advise clinically on this matter. More robust research is required (Table 4) to determine whether GHT mediates alterations in BP of transgender individuals, whether this change is associated with increased cardiovascular risk, and if such risk exists, what interventions can be undertaken to improve the health of transgender people.

**TABLE 4:** Gaps in evidence and recommendations for future research

Research question	Recommendations for future research
Does GHT alter the blood pressure of transgender men and/or women?	Interrupted time series or controlled cohort study measuring blood pressure before and after the introduction of GHT using validated office or ideally ambulatory blood pressure recordings
Are alterations in blood pressure associated with increased cardiovascular risk in transgender men and women?	Prospective longitudinal observational cohort study of transgender people for cardiovascular disease in relation to components of blood pressure
Do blood pressure-lowering interventions reduce cardiovascular risk in transgender men and women?	Randomized controlled study of transgender individuals with SBP > 130 mmHg to SBP targets of <120 mmHg (intensive treatment) or less than 140 mmHg (standard treatment) with a primary composite outcome of myocardial infarction, other acute coronary syndromes, stroke, heart failure or death from cardiovascular causes

GHT, gender-affirming hormone therapy.

## ACKNOWLEDGEMENTS

Previous presentations: Neither the whole work nor part of the work presented in this article has been previously presented.

The current work was supported by the British Heart Foundation (Centre of Research Excellence Awards RE/13/5/30177 and RE/18/6/34217).

### Conflicts of interest

There are no conflicts of interest.
